# Prolactin and Triiodothyronine Modulate Seasonal Variation in Brown Adipose Tissue in Djungarian Hamsters

**DOI:** 10.1002/jez.70103

**Published:** 2026-06-01

**Authors:** Sayantan Sur, Grace Payling, Calum Stewart, Avalene Tan, Caitlin Diggle, Tyler J. Stevenson

**Affiliations:** ^1^ School of Biodiversity, One Health & Veterinary Medicine University of Glasgow Glasgow UK; ^2^ The University of Texas at Dallas (UT Dallas) Dallas Texas USA

**Keywords:** adipose, liver, mammals, metabolism, photoperiod, rheostasis

## Abstract

Seasonal photoperiodic cycles regulate energy rheostasis in many animals. Brown adipose tissue (BAT) and the liver are key metabolic organs that show remarkable plasticity in response to photoperiodic conditions. Prolactin (PRL) and thyroid hormones are endocrine signals that transmit neural‐derived photoperiodic time measurement encoding to regulate peripheral tissues. Using Djungarian hamsters (*Phodopus sungorus*), a well‐established seasonal model, we examined the effects of photoperiod, triiodothyronine (T3), and PRL on liver and brown adipose tissue morphology and lipid content. Tissue mass, brown adipose tissue somatic index, and histological lipid content were quantified in three experiments that investigated tissue variation (1) across photoinduced seasonal cycles, and the sufficiency of acute (2) triiodothyronine, and (3) prolactin administration. Long photoperiod (LP) exposure maintained large BAT and higher lipid content, whereas short photoperiod (SP) exposure reduced BAT mass and lipid content. T3 treatment increased the BAT mass but did not impact lipid content. Conversely, PRL treatment increased BAT lipid content but did not impact tissue mass. Together, these findings identify triiodothyronine and prolactin as critical regulators of metabolic tissues.

## Summary

- Long photoperiod promoted brown adipose tissue hypertrophy and lipid accumulation in Djungarian hamsters.
- Prolactin increased brown adipose tissue lipid accumulation.
- Triiodothyronine increased brown adipose somatic index without altering lipid accumulation.

## 1 Introduction

Animals in temperate climes use environmental cues to regulate seasonal energy rheostasis, marked by changes in body mass, metabolic rate, and thermoregulatory strategies (Ruf and Geiser 2015; Stevenson 2023). The annual change in daylength, or photoperiod, is the primary predictive cue that mammals encode and regulate seasonal physiology (Stevenson et al. 2022). In most animals, photoperiod entrains an endogenous circannual clock that regulates the seasonal period and timing of distinct physiological, behavioral, and morphological phases (Lincoln 2019). Some animals, such as African stonechats (*Saxicola torquatus*) and golden‐mantled ground squirrels (*Callospermophilus lateralis*), maintain circannual rhythms in seasonal physiology over multiple years despite the absence of photoperiodic variation (Gwinner and Dittami 1990; Pengelley and Fisher 1957). Other animals, such as Djungarian hamsters (*Phodopus sungorus*), exhibit circannual timers in which a period of dormancy develops (e.g., 6 months) in response to short photoperiod (SP), but do not exhibit annual rhythms and instead display refractoriness to photoperiodic cues (Bittman 1978). These seasonal adaptations in physiological timing are important to optimize energy storage and utilization via remodeling of key metabolic tissues, such as the adipose tissue and liver (Navarro‐Masip et al. 2023). In mammals, brown adipose tissue (BAT) acts as a specialized thermogenic organ that dissipates stored energy as heat via non‐shivering thermogenesis in response to thermal and metabolic demands (Ballinger and Andrews 2018). In Djungarian hamsters (*Phodopus sungorus*), the BAT shows robust photoperiod‐dependent plasticity, in which the tissue mass and lipid content are highest during summer‐long photoperiods (LP) and decline during winter‐SP (Marshall et al. 2024; Rafael et al. 1985). Despite the reduction in tissue mass, the thermogenic capacity of the BAT is substantially upregulated during winter acclimatization to support increased heat production (Rafael et al. 1985). In Djungarian hamsters, the liver shows distinct seasonal changes, with SP exposure increasing hepatic fibroblast growth factor 21 (FGF21) production, aiding energy rheostasis during winter (Samms et al. 2014). Conversely, hepatic mitochondria undergo an active reduction in state 3 and state 4 respiration, thereby decreasing metabolic output during daily torpor (Kutschke et al. 2013).

Photoperiod‐regulated changes in thyroid hormones and prolactin are essential for seasonal changes in peripheral tissues (Appenroth and Cázarez‐Márquez 2024; Stewart and Marshall 2022). Circulating thyroid hormones (i.e., triiodothyronine, T3, and thyroxine, T4) act as metabolic regulators, increasing basal metabolic rate and modulating energy balance across tissues (Mullur et al. 2014). Thyroid hormones directly regulate BAT thermogenesis by enhancing the expression of thermogenic genes such as the uncoupling protein 1 (*Ucp1*) and stimulate mitochondrial activity to increase metabolic rate (Bianco and McAninch 2013). Thyroid hormones further govern liver function by regulating hepatic lipid and glucose metabolism, mitochondrial activity, and cholesterol homeostasis through thyroid hormone receptor–mediated gene regulation (Sinha et al. 2025). In Djungarian hamsters, winter short photoperiod reduces circulating T4 levels compared to summer LP (Masuda and Oishi 1989). However, experimentally restoring circulating T4 to summer LP levels under short photoperiod exposure failed to prevent seasonal reductions in body mass, adipose tissue mass, or reproductive regression (O'jile and Bartness 1992). Exogenous T3 administration in SP‐housed Djungarian hamsters induced a rapid decrease in peripheral leukocytes and stimulated gonadal development (Banks et al. 2016; Freeman et al. 2007). Moreover, T3 injections significantly reduced body mass in both LP‐ and SP‐housed hamsters, indicating photoperiod‐dependent peripheral responsiveness to triiodothyronine hormone (Banks et al. 2016).

Other circulating hormones, such as prolactin (PRL), are another rheostatic endocrine signal that drives long‐term photoperiod‐driven changes in body mass, food intake, adipose tissue function, and thermoregulatory physiology (Stewart and Marshall 2022). In Djungarian hamsters, exposure to SP suppresses circulating PRL levels compared to LP conditions (Bartness et al. 1987). Further, suppression of PRL release via bromocriptine reduces body mass to winter‐like levels, whereas PRL administration is sufficient to induce sustained body mass gain in Djungarian hamsters (Marshall et al. 2024). In golden hamsters (*Mesocricetus auratus*), hyperprolactinemia prevents the SP‐induced increases in BAT mass and thermogenic capacity (Kott et al. 1989). Transcriptomic profiling in Djungarian hamsters suggests that both BAT and liver express thyroid hormone receptors (*Thra*/*Thrb*) and the prolactin receptor (*Prlr*), indicating the capacity of the circulating hormones to act directly on these peripheral tissues (Stewart et al. 2022). Taken together, both thyroid hormones and PRL exhibit photoperiodic regulation and are potentially crucial mediators of seasonal energy rheostasis in mammals.

The current study aimed to understand the seasonal variation in lipid content in metabolic tissues using the Djungarian hamster. Hamsters exhibit robust, photoperiod‐driven changes in body mass, energy balance, thermoregulation, and reproductive physiology under captivity that closely mirror natural seasonal adaptations (Bao et al. 2019; Goldman 2002). Three experiments were conducted to examine the impact of photoperiod, triiodothyronine, and prolactin on brown adipose tissue and liver physiology by measuring tissue mass and lipid content. Experiment 1 used well‐characterized photoperiodic manipulation to study seasonal changes in hepatic and brown adipose tissue lipid content. Experiment 2 investigated the sufficiency of triiodothyronine (T3) to increase lipid content in short photoperiod acclimatized hamsters following acute (1‐day) and short‐term (7‐day) hormone administration. Lastly, experiment 3 was conducted to assess the acute effects of PRL as a long photoperiod signal to facilitate lipid content in hepatic and brown adipose tissue. Overall, these findings highlight PRL as a key photoperiodic endocrine signal that conveys seasonal information to peripheral metabolic organs.

## 2 Materials and Methods

### 2.1 Ethical Approval and Animal Maintenance

The experiments were performed under the UK Home Office Project License (PP5701950) with approval from the University of Glasgow Animal Welfare and Ethics Review Board. Adult Djungarian hamsters (*Phodopus sungorus*) were group‐housed at the Veterinary Research Facility, University of Glasgow, and kept on a long photoperiod (LP; 16 light: 8 dark). Animals had ad libitum access to food (rodent chow, Harlan Teklad) and water.

### 2.2 Experiments

Three studies were conducted to assess the endocrine regulation of histological and lipid content in hepatic and brown adipose tissues. Exposure to long photoperiod reinstates estrous cyclicity in female Djungarian hamsters and is associated with elevated and variable circulating prolactin levels relative to males (Ebling 1994; Lynch et al. 2016). To preclude this confounding effect, experiments 1 and 2 employed male hamsters to investigate seasonal metabolic changes. Conversely, Experiment 3 was performed in female hamsters maintained under short photoperiod conditions due to our previous observation of robust responses to circulating prolactin (Marshall et al. 2024).

#### 2.2.1 Experiment 1: Photoinduced Seasonal Changes in Metabolic Tissues

To understand seasonal adaptations in hepatic and brown adipose tissues, hamsters were exposed to a simulated photoregulated seasonal rhythm. The experimental design and tissues sampled in this study were obtained from hamsters used in a previous publication (Stewart et al. 2025). In brief, adult male hamsters (*N* = 30) were housed under LP (16 L:8D) conditions. A subset of animals (*N* = 6) remained under LP throughout the study and served as a reference group. The remaining hamsters were transferred to SP conditions (8 L:16D). At 8‐week intervals, subsets of SP‐exposed hamsters (*N* = 6 per time point) were collected following 8, 16, 24, and 32 weeks. Interscapular BAT was collected and weighed on a Sartorius cp64 anatomical balance (0.0001 g). Brown adipose somatic index (BASI) was calculated using the formula (BAT mass/body mass) × 100. The BAT and liver were stored at −80°C until histological assays.

#### 2.2.2 Experiment 2: Investigating the Role of T3 in Seasonal Metabolic Adaptation

Adult male hamsters (*N *= 27) were used to investigate the role of T3 in regulating lipid content in hepatic and brown adipose tissue. A subset of animals (*N *= 5) remained under LP and served as an LP reference group. The rest of the animals were transferred from LP to SP conditions for 12 weeks (*N *= 22) to initiate and establish the circannual maintenance phase (Stewart et al. 2025), and a subset (*N* = 8) served as the SP reference group. During the final week of SP exposure, hamsters were pseudorandomly divided into two treatment groups. One group received daily subcutaneous injections of triiodothyronine (5 µg/100 µL; Merck, 102467157) for 7 days (TH7; *n* = 7), whereas a second group received subcutaneous injections of 0.9% (w/v) saline for 6 days followed by a single triiodothyronine injection (5 µg/100 µL) prior to lights‐off on the final night (TH1; *n* = 7). The following day, hamsters were euthanized, and the interscapular BAT and liver were collected and stored at −80°C.

#### 2.2.3 Experiment 3: Investigating the Role of PRL in Seasonal Metabolic Adaptation

Adult female hamsters (*N *= 11) were used to assess the sufficiency of circulating PRL in driving seasonal change in hepatic and brown adipose tissues. The hamsters in this experiment were used in a previous publication (Marshall et al. 2024). Female hamsters were maintained under LP conditions and subsequently transferred to SP conditions for 8 weeks. Animals were then pseudorandomly assigned to one of two treatment groups. A control group received intraperitoneal injections of vehicle (100 µL; SAL: 50% dimethyl sulfoxide and 50% saline) for 1 day (*n *= 5). The treatment group received prolactin (PRL; 18 µg, NIAMDD‐O‐PRL) dissolved in vehicle and administered via intraperitoneal injections (100 µL) for 1 day (*n *= 6) (Marshall et al. 2024). All injections were performed during the mid‐light phase. The following day, hamsters were euthanized, and the interscapular BAT and liver were collected and stored at −80°C.

### 2.3 Histological Assays

#### 2.3.1 Hematoxylin and Eosin Staining of the BAT

**Figure 1:**
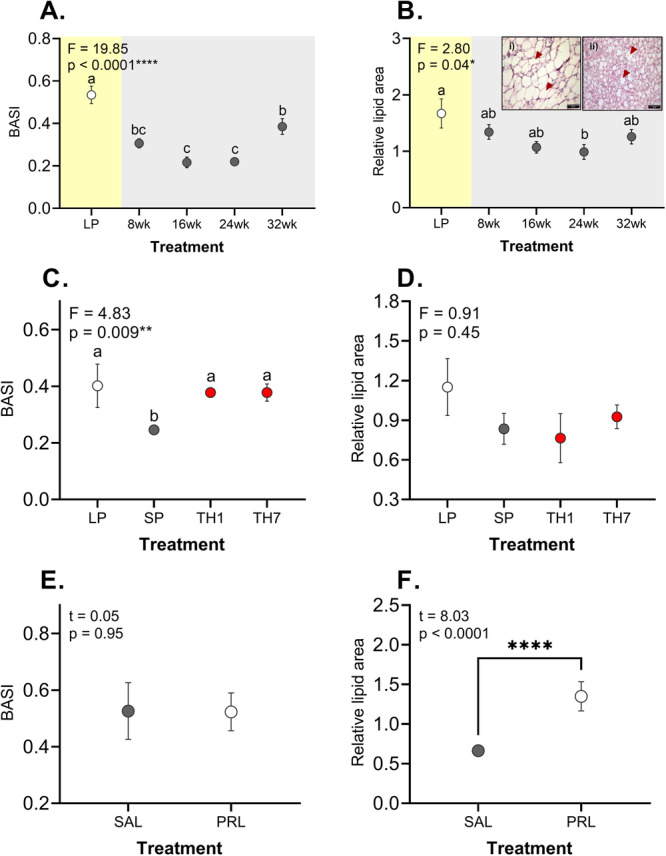
Seasonal changes in brown adipose tissue (BAT) morphometry and histology. Experiment 1. Mean ± SEM (*N* = 6, each group) values of the brown adipose somatic index, BASI (A), and relative lipid area (RLA) (B) across phototreatment groups (long photoperiod, LP; short photoperiod, SP8–32 weeks). Histological sections of BAT stained with hematoxylin and eosin (H&E) in LP (B‐i) and SP24wk (B‐ii). Red arrowheads indicate clear areas corresponding to lipid droplets. Scale bar = 100 µm. Experiment 2. Mean ± SEM values of BASI (C), and relative lipid area (RLA) (D) in LP (BASI, *N* = 5; RLA, *N* = 3), SP (BASI, *N* = 8; RLA, *N* = 8), Triiodothyronine Day 1 (TH1) (BASI, *N* = 7; RLA, *N* = 5), and Triiodothyronine Day 7 (TH7) (BASI, *N* = 7; RLA, *N* = 4) treatment groups. Experiment 3. Mean ± SEM values of BASI (E) and relative lipid area (RLA) (F) in saline (SAL, *N* = 5) and Prolactin (PRL, *N* = 6) treatment groups. Sample sizes (*N*) vary across groups due to occasional tissue loss during histological processing. Letters and asterisks represent significant differences in mean values. Statistical significance was determined at *p* ≤ 0.05. *p ≤ 0.05, **p ≤ 0.01, ****p ≤ 0.0001.

Hematoxylin and Eosin (H&E) staining was performed to identify seasonal and hormone‐dependent changes in BAT morphology. Frozen BATs were cryosectioned at 12 μm thickness on a Leica CM1850 cryostat, and from each animal, five sections were stained with H&E stain following the protocol of a previous study (Sur et al. 2025). Brightfield images acquired using a Leica DM4000B microscope (10× eyepiece * 20× objective magnification) were analyzed using ImageJ software. Lipid content across the entire surface area was quantified as the proportion of clear areas relative to stained cellular regions to determine the relative lipid area (Khaibullina et al. 2012) (see Figure 1).

#### 2.3.2 Oil Red O Staining of the Liver

**Figure 2:**
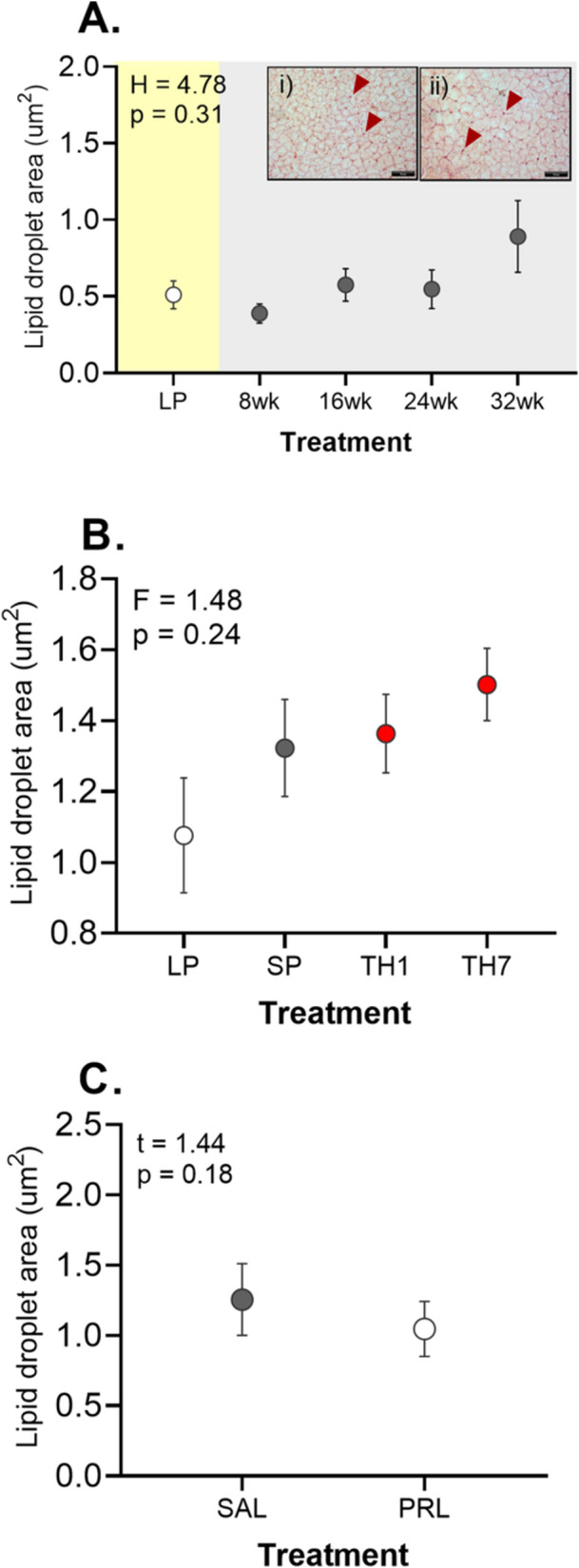
Seasonal changes in liver histology. Experiment 1. Mean ± SEM (*N* = 6, each group) values of hepatic lipid droplet area (µm^2^) (A) across phototreatment groups (long photoperiod, LP; short photoperiod, SP8–32 weeks). Histological sections of liver stained with Oil Red‐O (ORO) in LP (A‐i) and SP24wk (A‐ii). Red arrowheads indicate lipid droplets. Scale bar = 50 µm. Experiment 2. Mean ± SEM values of hepatic lipid droplet area (µm^2^) (B) in LP (*N* = 4), SP (*N* = 8), Triiodothyronine Day 1 (TH1; *N* = 7), and Triiodothyronine Day 7 (TH7; *N* = 7) treatment groups. Experiment 3. Mean ± SEM values of hepatic lipid droplet area (µm^2^) (C) in Saline (SAL, *N* = 5) and Prolactin (PRL, *N* = 5) treatment groups. Sample sizes (*N*) vary across groups due to occasional tissue loss during histological processing. Statistical significance was determined at *p* ≤ 0.05.

Lipid accumulation in the liver was assessed using Oil Red O (ORO) staining. Frozen liver samples were cryosectioned at 12 µm thickness using a Leica CM1850 cryostat, and from each animal, five sections were stained with ORO stain following the protocol from a previous study (Sur et al. 2025). Bright‐field images were acquired using a Leica DM4000B microscope (10 × eyepiece * 40× objective) and analyzed using ImageJ software (Deutsch et al. 2014; Sur et al. 2019). Images were converted to 8‐bit grayscale and subsequently binarized. Individual lipid droplets were separated using the watershed function and quantified using the analyze particles tool. For each image, the area (µm^2^) of 100 lipid droplets was measured and averaged to estimate lipid droplet area (LDA) for each animal (Sur et al. 2025, 2019) (see Figure 2).

### 2.4 Statistical Analyses

Normality of the data was evaluated using the Shapiro–Wilk test, and subsequent analyses were conducted using either parametric or non‐parametric tests as appropriate. In experiment 1, a one‐way ANOVA was performed on BAT mass, BASI, and relative lipid area; a Kruskal–Wallis test was performed on hepatic LDA. In experiment 2, a one‐way ANOVA was performed on BASI, relative lipid area, and hepatic LDA; a Kruskal–Wallis test was performed on BAT mass. The one‐way ANOVA was followed by Bonferroni's posttest, and the Kruskal–Wallis test was followed by Dunn's posttest for multiple comparisons. In experiment 3, an unpaired Student's *t*‐test was performed on BAT mass, BASI, relative lipid area, and hepatic LDA. For one‐way ANOVA, Kruskal–Wallis test, and unpaired Student's *t*‐test, effect sizes were calculated using eta‐squared (*η* ^2^), epsilon‐squared (*ε* ^2^), and Cohen's *d*, respectively (Tiwari et al. 2023; Tomczak and Tomczak 2014). A summary table of statistical analyses has been provided as Table 1. All statistical tests were considered significant at *p* ≤ 0.05.

**Table 1:** Summary of statistical analyses.

A: Experiment 1			
Parameters	*F*‐/*H*‐value	*p* value	*η* ^2^/*ε* ^2^
BAT mass	*F* = 22.35	< 0.0001	*η* ^2^ = 0.78
BASI	*F* = 19.85	< 0.0001	*η* ^2^ = 0.76
RLA	*F* = 2.80	0.04	*η* ^2^ = 0.30
Hepatic LDA	*H* = 4.78	0.31	*ε* ^2^ = 0.03

B: Experiment 2			
Parameters	*F*‐/*H*‐value	*p* value	*η* ^2^/*ε* ^2^
BAT mass	*H* = 11.60	0.0089	*ε* ^2^ = 0.37
BASI	*F* = 4.83	0.0094	*η* ^2^ = 0.38
RLA	*F* = 0.91	0.45	*η* ^2^ = 0.14
Hepatic LDA	*F* = 1.48	0.24	*η* ^2^ = 0.16

C: Experiment 3			
Parameters	*t*‐value	*p* value	Cohen's *d*
BAT mass	1.75	0.11	1.17
BASI	0.05	0.95	0.03
RLA	8.03	< 0.0001	5.35
Hepatic LDA	1.44	0.18	1.02

*Note:* Small (*η* ^2^/*ε* ^2^ ≥ 0.01; *d* ≥ 0.2), medium (*η* ^2^ ≥ 0.06, *ε* ^2^ ≥ 0.08; *d* ≥ 0.5), large (*η* ^2^ ≥ 0.14, *ε* ^2^ ≥ 0.26; *d* ≥ 0.8) effect.

## 3 Results

### 3.1 BAT Lipid Content in Response to Photoperiod and Hormone Treatment

One‐way ANOVA revealed a significant effect of photoperiod on BAT mass (Figure S1A; *η* ^2^ = 0.78; *F* = 22.35, *p*< 0.0001), BASI (Figure 1A; *η* ^2^ = 0.76; *F *= 19.85, *p*< 0.0001), and relative lipid area (Figure 1B; *η* ^2^ = 0.30; *F* = 2.80, *p* = 0.04). Compared to LP, BAT mass was reduced in SP 8‐, 16‐, and 24‐week hamsters (*p* ≤ 0.05, Bonferroni's posttest). By 32 weeks, there was a significant increase compared to 16‐ and 24‐week hamsters (*p* ≤ 0.05, Bonferroni's posttest). Exposure to SP for 24 weeks significantly reduced the relative lipid area compared to LP (*p* ≤ 0.05, Bonferroni's posttest). There was no significant difference between LP and 8‐, 16‐ or 32‐week hamsters (*p* > 0.05).

There was a significant one‐way ANOVA across treatment groups for BASI (Figure 1C; *η* ^2^ = 0.38; *F* = 4.83, *p*= 0.009). SP treatment significantly reduced BASI compared to LP and triiodothyronine‐treated hamsters (*p *≤ 0.05, Bonferroni's posttest). There was no significant variation in the relative lipid area across treatment groups (Figure 1D; *p*> 0.05, one‐way ANOVA). A Kruskal–Wallis test revealed triiodothyronine treatment had a significant impact on BAT mass (Figure S1B; *ε* ^2^ = 0.37; *H* = 11.60, *p* = 0.008). BAT mass was significantly lower in SP compared to LP, TH1, and TH7 (*p* ≤ 0.05, Dunn's posttest).

Prolactin treatment did not impact BAT mass or BASI (Figure S1C, Figure 1E; *p*> 0.05, Student's *t*‐test). Student's *t*‐test indicated that prolactin treatment significantly increased the relative lipid area compared to vehicle‐treated hamsters (Figure 1F; *d* = 5.35; *t* = 8.03, df = 9, *p* < 0.0001).

### 3.2 Hepatic Lipid Content in Response to Photoperiod and Hormone Treatment

A Kruskal–Wallis test revealed no significant effect of photoperiod on the hepatic lipid content (Figure 2A; *p*> 0.05). One‐way ANOVA revealed no changes in hepatic LDA in response to triiodothyronine treatment (Figure 2B; *p*> 0.05). Furthermore, prolactin treatment was not observed to regulate hepatic lipid content (Figure 2C; *p* > 0.05, Student's *t*‐test).

## 4 Discussion

**Figure 3:**
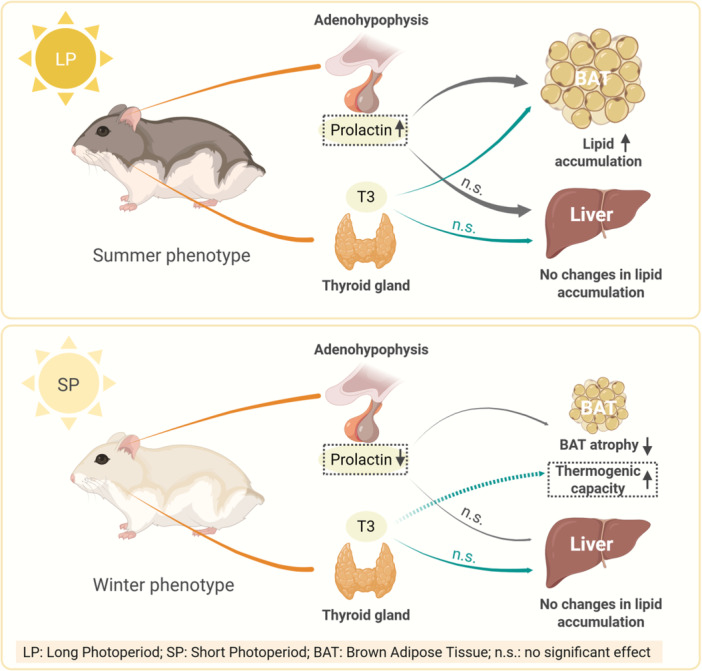
Schematic illustrating a programmed rheostatic model of seasonal regulation of brown adipose tissue (BAT) in Djungarian hamsters. Upper panel. Summer‐long photoperiod (LP) induces prolactin (PRL) secretion from the adenohypophysis, contributing to BAT hypertrophy and lipid accumulation. BAT exhibits reduced thermogenic activity, and the stored lipids function as a readily mobilizable fuel reserve. Lower panel. Winter‐short photoperiod (SP) reduces circulating PRL levels, contributing to BAT atrophy. Triiodothyronine (T3), secreted by the thyroid gland, increases the thermogenic capacity of the BAT, thereby supporting winter acclimatization. Hepatic lipid accumulation remains unchanged across seasons, suggesting a protective mechanism against hepatosteatosis. The dashed lines and boxes indicate causal relationships inferred from the literature.

The current study demonstrates robust photoperiodic and endocrine regulation of BAT physiology in Djungarian hamsters and highlights prolactin as a key seasonal signal driving tissue remodeling across annual cycles (Figure 3). Exposure to LP maintains larger BAT and increased lipid accumulation, whereas SP was associated with reduced BAT mass and lipid content, consistent with winter adaptations in hamsters. Despite pronounced seasonal changes in BAT morphology, hepatic lipid accumulation remained unchanged across photoperiods and hormonal treatment, indicating tissue‐specific mechanisms against steatotic conditions.

### 4.1 Seasonal Adaptations in BAT Physiology

Photoperiodic manipulation reduced BAT mass, BASI, and relative lipid area in Djungarian hamsters (Figure 1, Figure S1). This is consistent with previous studies in hamsters, showing that during summer growth, BAT comprises nearly 5% of body mass, with lipids accounting for approximately 86% of total BAT mass (Rafael et al. 1985). During summer‐LP conditions, circulatory PRL levels peak in both male and female hamsters (Ebling 1994). Our findings indicate that increased PRL secretion conveys a summer photoperiodic signal that is sufficient for lipid accumulation in BAT (Figure 1F; Figure 3). This PRL‐induced lipid deposition likely reflects reduced thermogenic demand and a shift toward energy storage associated with positive energy balance during summer. The BAT shows a marked decrease in mass and lipid content following exposure to winter‐SP conditions (Figure 1A,B, Figure S1). Although BAT mass is reduced, mitochondrial protein content in BAT increases by ~four‐fold under winter conditions (Rafael et al. 1985). Further, cytochrome c oxidase activity increases by up to 700%, depending on the specific BAT fat pad, during winter (Rafael et al. 1985). In Djungarian hamsters, circulating T3 levels increase at lower ambient temperatures (7°C > 25°C) and are not significantly affected by photoperiod (Masuda and Oishi 1989). Likewise, under natural photoperiodic conditions, circulating total and free T3 levels increase during winter in adult Djungarian hamsters (Seidel et al. 1987). T3 stimulates thermogenesis in BAT by promoting mitochondrial uncoupling of electron transport from ATP synthesis (Yau et al. 2018). It is therefore likely that under winter‐SP conditions, T3 contributes to enhanced thermogenic activity through uncoupled mitochondrial respiration and the expression of thermogenic *Ucp1* gene in Djungarian hamsters (Yau et al. 2018) (Figure 3). This is supported by our data, showing that T3 significantly increased the cellular content of BAT without altering lipid accumulation (Figure 1C,D). Future studies validating changes in thermogenic markers at the transcript and protein level will be critical for understanding the mechanisms regulating seasonal BAT rheostasis, which is a limitation of the current study.

### 4.2 Hepatic Lipid Accumulation Remains Stable Across Seasons

Lipid accumulation in the liver remained unchanged across phototreatment groups in the hamsters (Figure 2A). These findings are common with Nile grass rats (*Arvicanthis niloticus*), where exposure to SP did not impact hepatic steatosis (Shankar et al. 2024). The liver plays a central role in lipid metabolism, controlling uptake, storage, synthesis, catabolism, and transport of lipids as part of systemic lipid balance (Duan et al. 2025). In mammals, several cellular pathways prevent hepatic triglyceride overload, including peroxisome proliferator‐activated receptor alpha (PPARα)‐driven fatty‐acid (FA) β‐oxidation, very low‐density lipoprotein (VLDL)–triglyceride export, and autophagy‐mediated lipid droplet clearance (Minehira et al. 2008; Singh et al. 2009; Todisco et al. 2022). Similar to Djungarian hamsters, Japanese quails (*Coturnix japonica*) maintain stable hepatic lipid stores across simulated seasonal cycles and upregulate markers associated with obesity resistance during vernal adipogenesis (Sur et al. 2025). In our study, treatment with T3 and PRL did not alter hepatic lipid accumulation (Figure 2B,C). PRL signaling can ameliorate hepatic lipid accumulation through modulation of FA transport pathways, such as cluster of differentiation 36 (CD36) (Zhang et al. 2018). While in rodents, T3 modulates hepatic lipid metabolism by promoting both lipid catabolism and lipogenesis via TH‐receptor activity (Senese et al. 2017). Previous studies in SP hamsters have shown that a single T3 injection is sufficient to alter hypothalamic somatostatin (*Sst*) expression compared to vehicles, supporting the effectiveness of the acute T3 regimen (Stewart et al. 2025). However, although short‐term T3 exposure is sufficient to induce transcriptional responses, the duration may be insufficient for these changes to translate into detectable lipid remodeling. In the current study, circulating hormone levels were not assessed, limiting inference of the direct contribution. Further, the low sample size in hepatic histology may have reduced statistical power, potentially contributing to the absence of detectable differences (Table 1).

## 5 Conclusion

Our findings show that photoperiodic information conveyed via endocrine signals drives seasonal remodeling of BAT structure in Djungarian hamsters. Summer‐LP conditions promoted BAT hypertrophy, while winter‐SP exposure induced a transient reduction followed by spontaneous recovery of BAT dimensions, consistent with photoperiodic regulation of metabolic processes and the development of photorefractoriness (Stewart et al. 2025). This transition marks a shift in photosensitivity, where prolonged photoperiod exposure attenuates tissue responsiveness despite sustained photic signaling. PRL encodes and signals photic information from the adenohypophysis to peripheral organs, thereby directing seasonal changes in tissue growth, metabolism, and energy rheostasis (Figure 3) (Stewart and Marshall 2022). This aligns with an earlier Djungarian hamster study showing that PRL mediates seasonal changes in renal morphology and transcriptional architecture (Sur et al. 2025). Circulating T3, in contrast, potentially promotes thermogenic activity in BAT as part of winter cold adaptation in hamsters (Figure 3). Notably, sex can influence the regulation of seasonal energetics, and the variable inclusion of both male and female cohorts may constrain comparisons across experiments. The liver maintains lipid homeostasis across seasons, thereby limiting excessive hepatic lipid accumulation. The independent roles of PRL and T3 in regulating seasonal energy rheostasis can be further delineated using factorial designs and/or blockade experiments in future studies.

## Author Contributions

Conceptualization by S.S. and T.J.S. Data curation by G.P., S.S., and T.J.S. Formal analysis by A.T., C.D., C.S., G.P., and S.S. Funding acquisition by T.J.S. Investigation by G.P. and S.S. Methodology by S.S. and T.J.S. Project administration and supervision by T.J.S. Resources by T.J.S. Validation by S.S. and T.J.S. Visualization by S.S. Writing – original draft preparation by S.S. and T.J.S. Writing – review and editing by S.S. and T.J.S. All authors contributed critically to the draft and gave final approval for publication.

## Conflicts of Interest

The authors declare no conflicts of interest.

## Supporting information


**Figure S1:** Seasonal changes in brown adipose tissue (BAT) mass. Experiment 1. Mean ± SEM (*N* = 6, each group) values of the BAT mass (A) across phototreatment groups (Long photoperiod, LP; Short Photoperiod, SP8‐32 weeks). **Experiment 2.** Mean ± SEM values of BAT mass (B) in LP (*N* = 5), SP (*N* = 8), Triiodothyronine Day 1 (TH1; *N* = 7), and Triiodothyronine Day 7 (TH7; *N* = 7) treatment groups. **Experiment 3.** Mean ± SEM values of BAT mass (C) in saline (SAL, *N* = 5) and prolactin (PRL, *N* = 6) treatment groups. Letters and asterisks represent significant differences in mean values. Statistical significance was determined at *p* ≤ 0.05.

## Data Availability

The data that support the findings of this study are available from the corresponding author upon reasonable request.
