# Supplementary figures and images for: Prolactin and Triiodothyronine Modulate Seasonal Variation in Brown Adipose Tissue in Djungarian Hamsters
Source: J Exp Zool A Ecol Integr Physiol. 2026 Jun 1;345(7):710–8. doi: 10.1002/jez.70103 (PMC13353641; doi:10.1002/jez.70103)

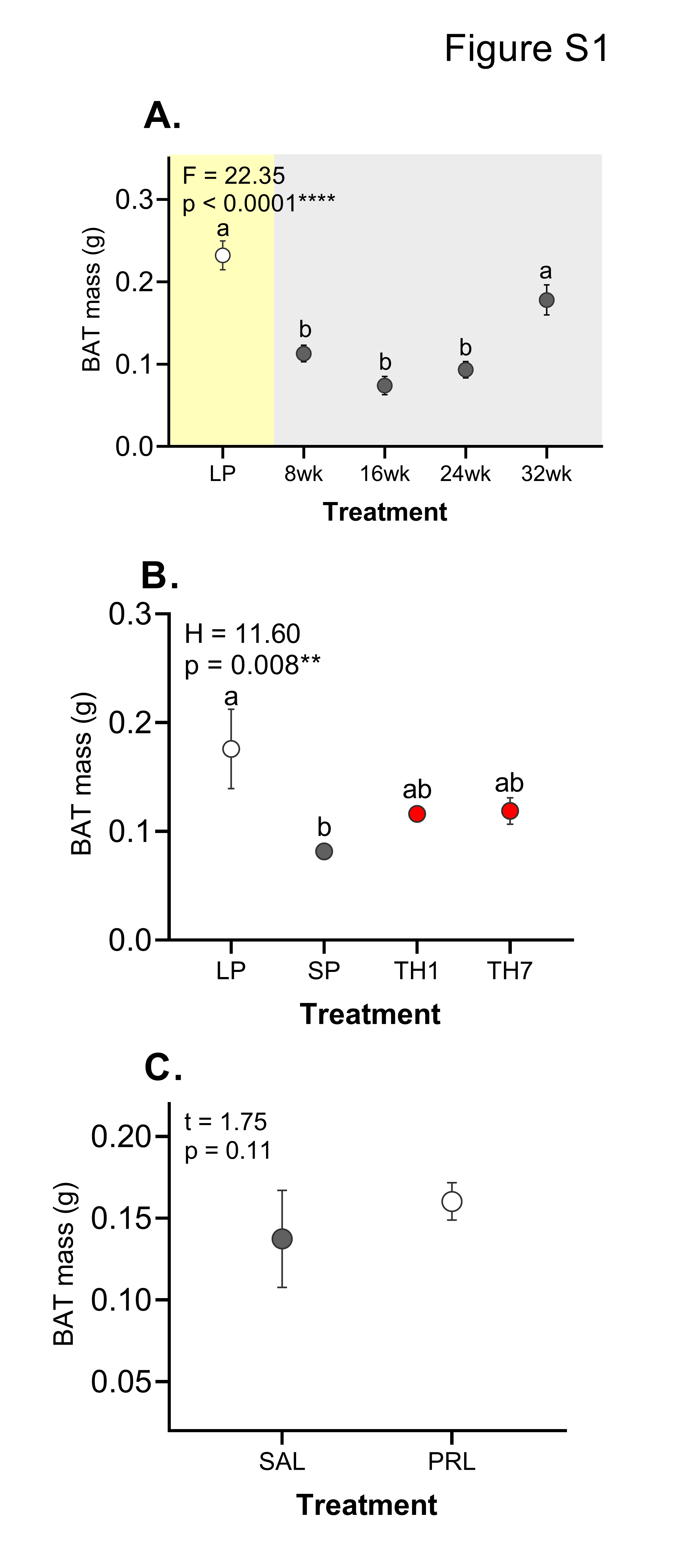

Supplement: Supplementary file 1 — Figure S1: Seasonal changes in brown adipose tissue (BAT) mass. Experiment 1. Mean ± SEM (N = 6, each group) values of the BAT mass (A) across phototreatment groups (Long photoperiod, LP; Short Photoperiod, SP8‐32 weeks). Experiment 2. Mean ± SEM values of BAT mass (B) in LP (N = 5), SP (N = 8), Triiodothyronine Day 1 (TH1; N = 7), and Triiodothyronine Day 7 (TH7; N = 7) treatment groups. Experiment 3. Mean ± SEM values of BAT mass (C) in saline (SAL, N = 5) and prolactin (PRL, N = 6) treatment groups. Letters and asterisks represent significant differences in mean values. Statistical significance was determined at p ≤ 0.05. [file JEZ-345-710-s001.tif]
